# Physicochemical, Predicted ADME, and Structural Positioning of Azelaic Acid Within an Endocrine Oncology Reference Framework

**DOI:** 10.3390/ph19101634

**Published:** 2026-10-15

**Authors:** Elena-Daniela Jurj, Adrian Voicu, Daiana Colibășanu, Mirela Voicu, Mirela Nicolov, Lucreția Udrescu

**Affiliations:** 1 Center for Drug Data Analysis, Cheminformatics, and the Internet of Medical Things, Victor Babeș University of Medicine and Pharmacy Timișoara, 300041 Timișoara, Romania; daniela.adam@umft.ro (E.-D.J.); daiana.handa@umft.ro (D.C.); mirelanicolov@umft.ro (M.N.); 2 Department II–Pharmacology, Physiology and Physiopathology, Victor Babeş University of Medicine and Pharmacy Timişoara, 300041 Timişoara, Romania; voicu.mirela@umft.ro; 3 Departament II–Pharmaceutical Chemistry, Victor Babeș University of Medicine and Pharmacy Timișoara, 300041 Timișoara, Romania; 4 Research Center for Experimental Pharmacology and Drug Design (X-Pharm Design), Victor Babeş University of Medicine and Pharmacy Timişoara, 300041 Timişoara, Romania; 5 Department I–Physical Chemistry and Pharmaceutical Physics, Victor Babeș University of Medicine and Pharmacy Timișoara, 300041 Timișoara, Romania; 6 Department I–Clinical Pharmacy and Drug Analysis, Victor Babeş University of Medicine and Pharmacy Timişoara, 300041 Timişoara, Romania

**Keywords:** azelaic acid, drug repurposing, endocrine anticancer drugs, SwissADME, principal component analysis, molecular similarity, Morgan fingerprints, Tanimoto similarity

## Abstract

**Background:** Azelaic acid, an established dermatologic dicarboxylic acid, was previously nominated as an unusual anticancer repurposing candidate. However, it remains unclear whether physicochemical properties, predicted ADME, or structural proximity to established endocrine anticancer drugs support this hypothesis. This study positioned azelaic acid relative to a clinically anchored endocrine anticancer reference panel using reference-defined space based on physicochemical and predicted ADME descriptors and two-dimensional structural similarity analyses. **Methods:** Sixteen clinically used endocrine anticancer drugs defined the reference set, with finasteride and dutasteride included as mechanistic comparators. SwissADME descriptors were screened for redundancy, standardized, and analyzed by principal component analysis and Euclidean nearest neighbor distances. Robustness was assessed by leave-one-reference-out, bootstrap, and WLOGP sensitivity analyses. Structural similarity was evaluated independently using Morgan fingerprints and Tanimoto coefficients. **Results:** The first two principal components explained 75.31% of reference variance. Flutamide was the nearest Clinical Reference to azelaic acid in physicochemical and predicted ADME space (*d* = 3.0063), but this distance exceeded the reference $q_{90}$ threshold (2.6521), classifying azelaic acid as extrapolative. This classification persisted in all 16 leave-one-reference-out analyses and in 73.69% of 992 valid bootstrap replicates. Structurally, the highest reference similarity was observed with toremifene (*T* = 0.08696), below the minimum internal Clinical Reference nearest-neighbor similarity (0.13761); radius-3 sensitivity analysis preserved this result. **Conclusions:** Azelaic acid is neither a close structural analog nor a physicochemical and predicted ADME neighbor of established endocrine anticancer drugs. Its network-derived nomination therefore represents a non-canonical repurposing hypothesis requiring further experimental validation.

## 1. Introduction

Endocrine manipulation remains a major therapeutic strategy in hormone-dependent malignancies, particularly estrogen receptor-positive breast cancer and androgen-driven prostate cancer [1]. Clinically used agents act at different levels of these pathways, including estrogen receptor modulation or degradation, aromatase inhibition, androgen receptor antagonism, inhibition of androgen biosynthesis, and suppression of gonadal androgen production [2,3]. These drugs therefore share a therapeutic context without necessarily sharing a common chemical scaffold or physicochemical profile; the endocrine oncology drug space encompasses structurally and pharmacokinetically heterogeneous compounds. Current breast and prostate cancer treatment resources continue to reflect this mechanistic diversity.

Drug repurposing provides an opportunity to identify therapeutic hypotheses beyond established chemotypes, particularly when candidate prioritization exploits biological, pharmacological, or network relationships instead of direct structural similarities [4,5,6,7]. In previous work, we developed a target-based drug–drug similarity network in which drugs were connected according to shared drug-target relationships and organized into pharmacologically enriched communities. Using the ratio between betweenness and degree centrality as a prioritization criterion, azelaic acid was identified as a potential antineoplastic repurposing candidate [8]. As an established dermatologic dicarboxylic acid and not a conventional anticancer chemotype, azelaic acid represents an unusual, non-canonical repurposing hypothesis. In that study, the network-derived nomination was further examined by molecular docking, which predicted an interaction between azelaic acid and steroid 17α-hydroxylase/17,20-lyase (CYP17A1), with an interaction pattern similar to those observed for progesterone and abiraterone. This provided a target-specific computational rationale for considering azelaic acid in an endocrine-oncology context, while remaining a hypothesis requiring experimental validation. However, neither network-based nomination nor target-level docking establishes whether a candidate occupies a molecular property space comparable to that of clinically used anticancer agents. More broadly, network-based drug repositioning approaches are intended to narrow a large hypothesis space for subsequent computational or experimental evaluation rather than to provide evidence of efficacy by themselves [6,8,9,10,11].

Azelaic acid (nonanedioic acid) is a naturally occurring saturated nine-carbon dicarboxylic acid with established topical use in dermatology, but interest in its antitumor properties predates contemporary drug-repurposing approaches. Early experimental work showed time- and dose-dependent antiproliferative and cytotoxic effects against several tumor-derived models, including human and murine cutaneous melanoma, human choroidal melanoma, squamous carcinoma, and lymphoma- and leukemia-derived cell lines, while normal cells were generally less affected under comparable exposure conditions [12,13,14]. Human primary melanoma cultures were also reported to be susceptible to azelaic acid, and xenotransplantation studies provided additional in vivo evidence of reduced melanoma cell proliferation and tumor growth [15,16]. Mechanistic investigations implicated inhibition of mitochondrial oxidoreductases and DNA synthesis, competitive inhibition of thioredoxin reductase in melanoma cells, and effects on cellular redox homeostasis [17,18,19,20]. Of particular relevance to hormone-dependent malignancies, azelaic acid was also reported to inhibit 5α-reductase activity in human skin microsomal preparations, although this observation does not establish prostate-specific enzyme inhibition or anticancer efficacy [21]. Early clinical reports and case series described regression of lentigo maligna and selected primary malignant melanoma lesions after azelaic acid treatment, but these observations preceded modern controlled oncological trial standards and should therefore be regarded as exploratory evidence [22,23]. On the basis of this broader biological profile, prostate, breast, bladder, bronchial and hematological malignancies were subsequently proposed as potential areas for investigation; importantly, these were hypotheses and not demonstrations of therapeutic activity [24]. More recent studies have renewed interest in this possibility by showing antileukemic effects in acute myeloid leukemia models involving Akt/Jab1/thioredoxin, Notch, and Prdx/ROS-related pathways, while more recent work has provided additional melanoma-related mechanistic hypotheses [25,26,27,28]. Taken together, these findings provide a biologically plausible but still incomplete rationale for repurposing azelaic acid, particularly because direct evidence in hormone-dependent solid tumors remains limited.

The translational question is complicated by the biopharmaceutical characteristics of azelaic acid. Pharmacokinetic studies show that systemic exposure varies greatly with the route of administration and formulation. Traditional topical formulations have limited skin absorption, while alternative vehicles enhance penetration [29,30,31]. After oral intake, serum concentrations peak within 2–3 h and decline quickly, with a significant portion of the dose excreted unchanged in urine within 12 h [31,32]. Intravenous administration of the disodium salt produced substantially higher systemic concentrations [31,33,34], while the non-excreted fraction underwent mitochondrial β-oxidation [32]. These observations are relevant because many historical antiproliferative and enzyme inhibition experiments used relatively high azelaic acid concentrations, emphasizing that biological plausibility alone does not resolve questions of exposure, delivery, or pharmaceutical feasibility.

A key computational question remains: how does azelaic acid compare with a prespecified panel of established endocrine anticancer drugs in physicochemical properties, predicted ADME, and two-dimensional structural similarity? SwissADME provides an integrated structure-based framework for evaluating physicochemical properties, predicted pharmacokinetic behavior, drug-likeness, and medicinal chemistry characteristics [35]. However, evaluating azelaic acid in isolation would provide limited translational context, because individual physicochemical or ADME predictions do not indicate whether a network-nominated candidate falls within or outside the property space occupied by clinically established therapies. We therefore considered a prespecified panel of endocrine anticancer agents as a clinically anchored calibration framework. The comparison was not intended to imply mechanistic or structural equivalence; instead, it tested whether a repurposing hypothesis generated independently from chemical similarity was also accompanied by proximity in physicochemical, predicted ADME, or two-dimensional structural space.

Accordingly, the present study uses azelaic acid as an external query molecule and positions it against a prespecified set of 16 clinically used endocrine anticancer agents spanning breast/endometrial and prostate cancer therapy. The clinical reference compounds alone were used to define descriptor distributions, scaling parameters, descriptor selection, principal component axes, and empirical nearest neighbor thresholds, while azelaic acid was subsequently projected into this reference space. These 16 compounds were intended as a clinically anchored therapeutic-context panel and were not assumed to constitute a mechanistically homogeneous class. Finasteride and dutasteride were included separately as external mechanistic comparators representing the 5α-reductase axis. Their purpose was to test whether this historical mechanistic association was accompanied by broader physicochemical or structural proximity to azelaic acid. The stability of the physicochemical and ADME positioning was evaluated by leave-one-reference-out and bootstrap analyses. In parallel, Morgan fingerprints and Tanimoto coefficients were used to determine whether similarity in property space was accompanied by two-dimensional structural similarity. Accordingly, the analysis was designed to quantify the position of the network-derived candidate relative to an internally defined clinical reference neighborhood. It also assessed the stability of this positioning across complementary analytical representations, without implying a common mechanism of action or anticancer efficacy.

## 2. Results

### 2.1. Compound Set and Descriptor Selection

The final dataset comprised 19 compounds: 16 Clinical Reference endocrine anticancer agents, azelaic acid as the Query molecule, and finasteride and dutasteride as external Mechanistic Comparators. Molecular identities were verified across the structure and metadata files before analysis.

Ten continuous SwissADME descriptors were initially considered for multivariate analysis. None met the prespecified low-variation exclusion criteria. Correlation-based redundancy screening removed molar refractivity and topological polar surface area, leaving eight descriptors for the primary analysis: molecular weight, hydrogen bond acceptors, hydrogen bond donors, rotatable bonds, Fraction Csp^3^, Consensus Log P, estimated solubility (ESOL Log S), and synthetic accessibility. All subsequent reference space analyses were therefore based on this eight-dimensional descriptor set (Tables S2 and S3; Figure S1).

### 2.2. Univariate Physicochemical and Predicted ADME Positioning of Azelaic Acid

**Figure 1:**
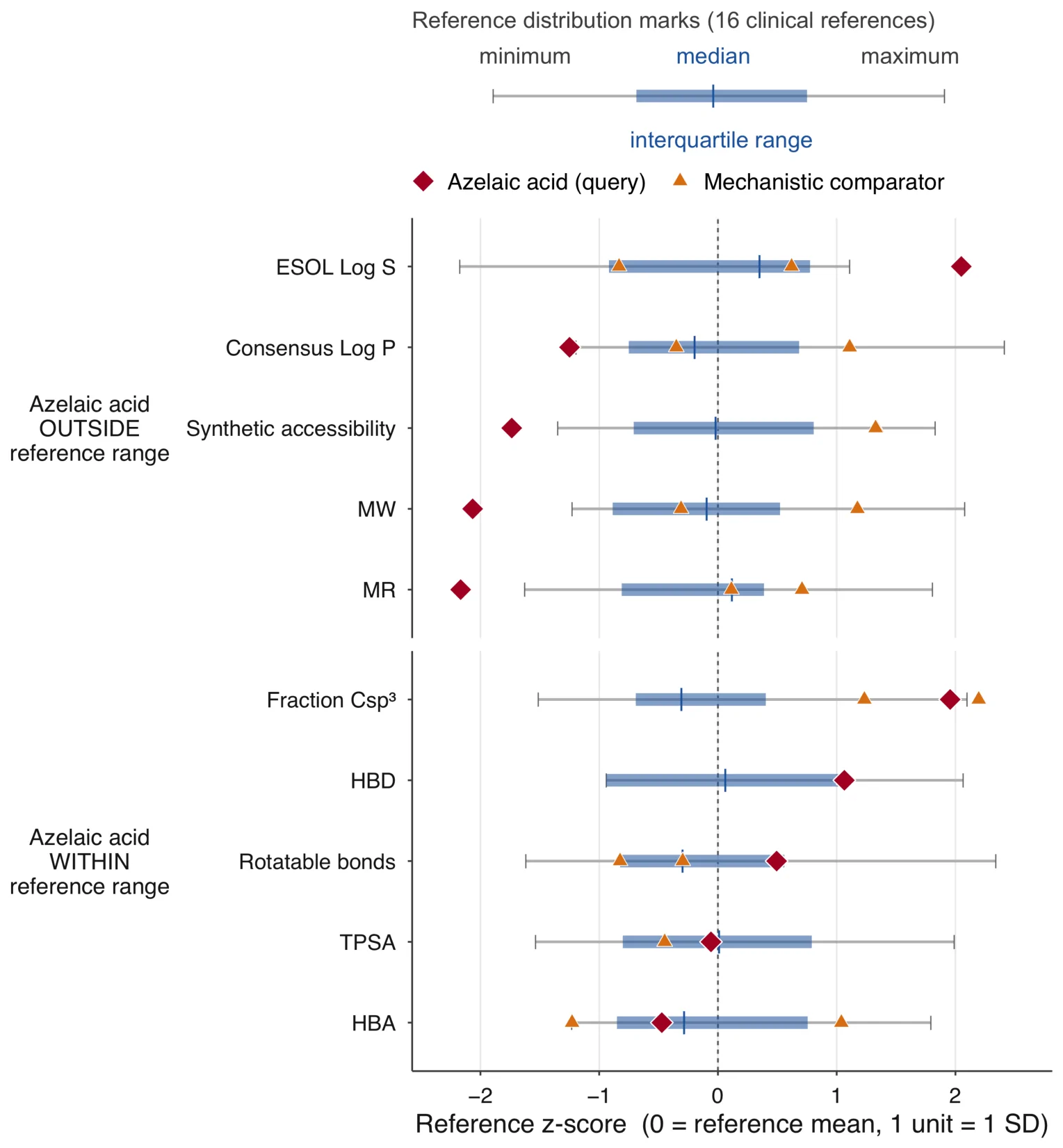
Univariate positioning of azelaic acid relative to the Clinical Reference distributions. Descriptor values were standardized using the mean and standard deviation of the 16 Clinical Reference compounds. Horizontal lines indicate the observed reference range, shaded bars the interquartile range, and vertical marks the reference median. Azelaic acid is shown as the Query molecule and finasteride and dutasteride as projected Mechanistic Comparators. Molar refractivity (MR) and topological polar surface area (TPSA) were included in the initial descriptor panel and are displayed for completeness but were excluded from subsequent multivariate modeling because of redundancy. A reference *z*-score of 0 corresponds to the Clinical Reference mean and one unit corresponds to one reference-set standard deviation.

Univariate comparison with the 16 Clinical Reference compounds showed that azelaic acid lay outside the observed reference range for five of the ten initially evaluated descriptors (Figure 1). Specifically, azelaic acid was positioned beyond the reference range for molecular weight (MW), molar refractivity (MR), Consensus Log P, ESOL Log S, and synthetic accessibility, whereas HBA, HBD, rotatable bonds, Fraction Csp^3^, and TPSA remained within the observed reference range. MR and TPSA are shown in Figure 1 for completeness but were subsequently excluded from multivariate modeling because of descriptor redundancy.

Among the eight descriptors retained for the primary analysis, azelaic acid had a molecular weight of 188.22 g/mol, below the minimum observed among the Clinical Reference compounds (276.21 g/mol; empirical percentile 0%; z=−2.07). Its SwissADME Consensus Log P was likewise below the reference range (1.50 vs. 1.61–8.75; percentile 0%; z=−1.25), whereas its predicted ESOL Log S was higher than that of every clinical reference (−1.47 vs. reference maximum −3.04; percentile 100%; z=2.05), indicating comparatively greater predicted aqueous solubility. The synthetic-accessibility score was also below the reference range (1.57 vs. 2.05–5.98; percentile 0%; z=−1.74), corresponding to lower predicted synthetic complexity.

By contrast, the hydrogen bond acceptor count (HBA = 4; percentile 40.6%; z=−0.47), hydrogen bond donor count (HBD = 2; percentile 81.3%; z=1.07), and number of rotatable bonds (8; percentile 71.9%; z=0.49) fell within the reference interquartile ranges. Fraction Csp^3^ was high relative to the Clinical Reference panel (0.78; percentile 93.8%; z=1.96) but remained within the observed reference range. Overall, the univariate profile therefore combined marked differences in molecular size, lipophilicity, predicted solubility, and synthetic accessibility with overlap in hydrogen-bonding capacity, conformational flexibility, and carbon saturation.

A detailed summary of the univariate positioning of azelaic acid across the eight retained descriptors is provided in Table 1, including the reference minimum–maximum range and IQR, empirical percentiles, reference-standardized *z*-scores, and relative position within the Clinical Reference distributions.

**Figure 2:**
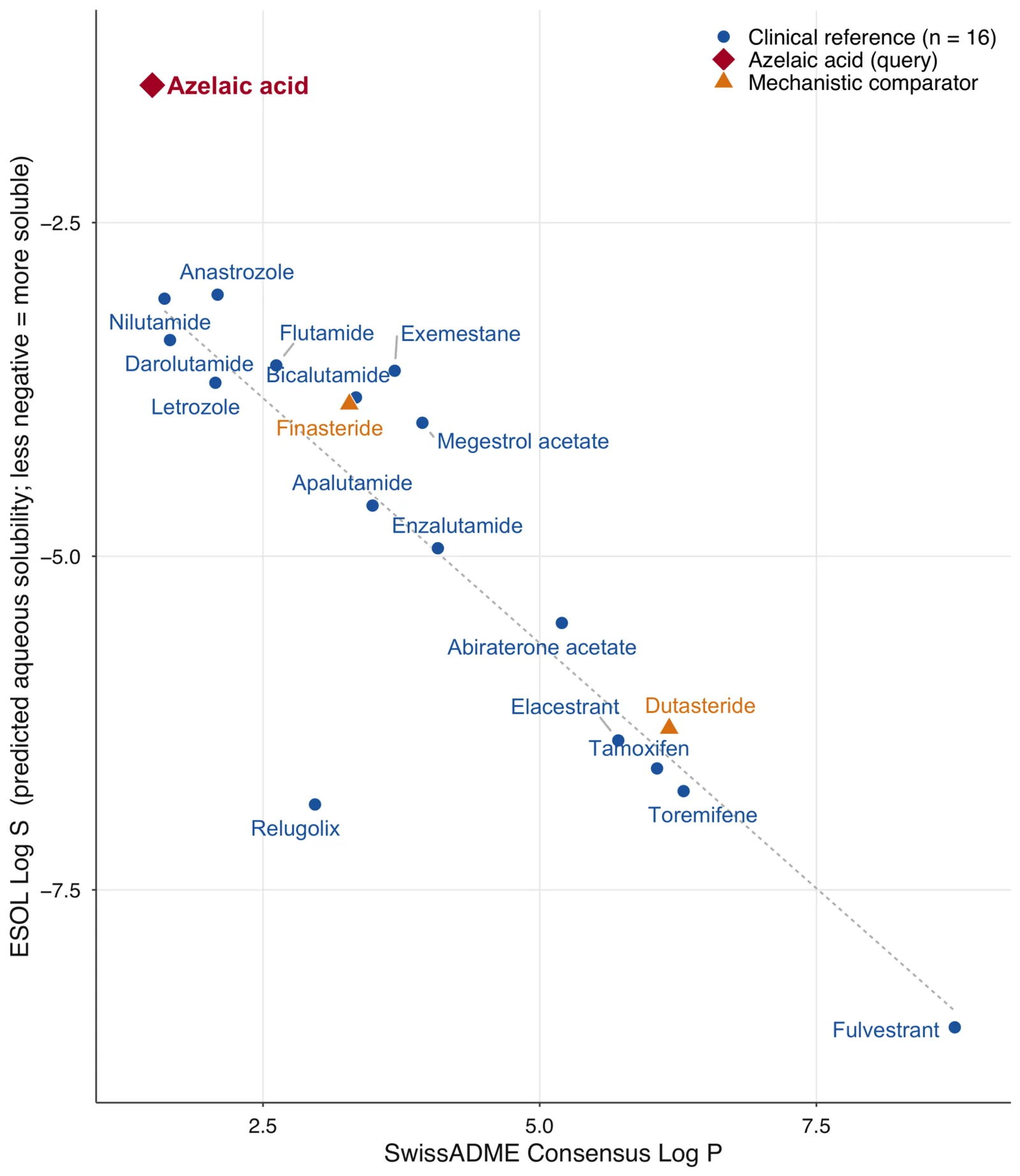
Joint positioning of azelaic acid by SwissADME Consensus Log P and ESOL Log S relative to the Clinical Reference set and Mechanistic Comparators. The dashed line represents the ordinary least-squares fit to the 16 Clinical Reference compounds only.

The joint lipophilicity-solubility representation further emphasized this separation: azelaic acid occupied the low-lipophilicity, high-predicted solubility region beyond the corresponding values of all 16 clinical references (Figure 2).

### 2.3. Principal Component Representation of the Clinical Reference Space

**Figure 3:**
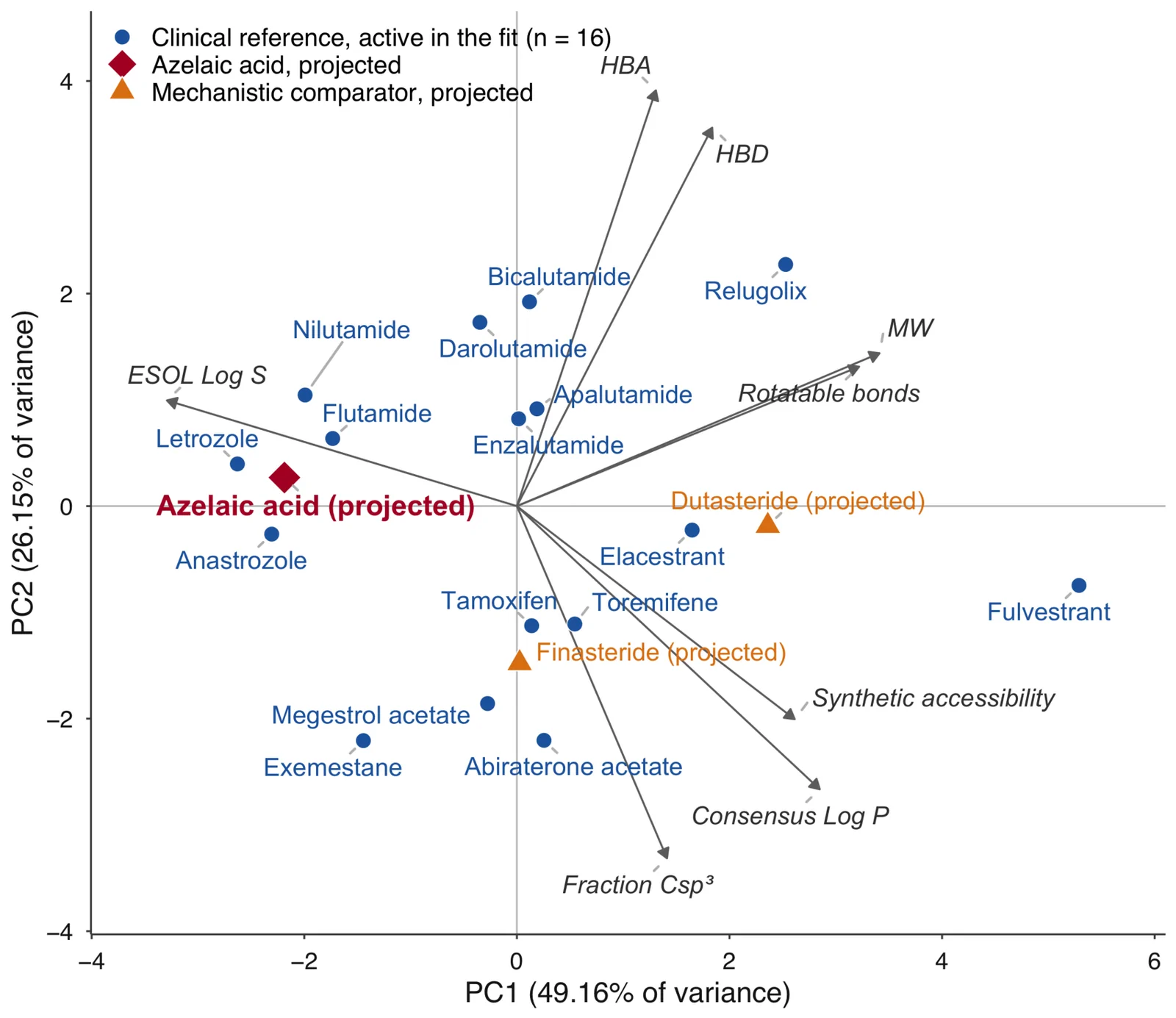
Reference-defined principal component analysis of the physicochemical and predicted ADME descriptor space. PCA was fitted exclusively to the 16 Clinical Reference compounds using the eight retained standardized descriptors. Azelaic acid (Query) and finasteride and dutasteride (Mechanistic Comparators) were subsequently projected using the reference-derived centering and scaling parameters and did not contribute to model fitting. PC1 and PC2 explained 49.16% and 26.15% of the reference variance, respectively (75.31% cumulative). Grey arrows indicate descriptor loadings and were scaled for graphical display. Nearest neighbor distances and empirical neighborhood classifications were calculated in the complete eight-dimensional standardized descriptor space and should not be inferred from distances in the two-dimensional PCA projection.

Principal component analysis was performed exclusively on the 16 Clinical Reference compounds after centering and scaling the eight retained descriptors. The first two principal components explained 75.31% of the variance in the clinical reference set (PC1, 49.16%; PC2, 26.15%; Figure 3; Table S4; Figures S2 and S3). Projection of azelaic acid into the fixed reference-derived PCA model positioned it toward the negative PC1 region of the reference space, opposite the direction of higher molecular size and lipophilicity. Finasteride and dutasteride occupied distinct projected positions and did not overlap with azelaic acid in the PC1-PC2 plane. Because PCA was used only for descriptive visualization [36], separation in the PC1-PC2 projection was not interpreted as evidence of molecular dissimilarity. Molecular proximity and neighborhood classification were assessed exclusively in the complete eight-dimensional standardized descriptor space.

### 2.4. Categorical ADME, Drug-Likeness, and Medicinal Chemistry Profile of Azelaic Acid

**Figure 4:**
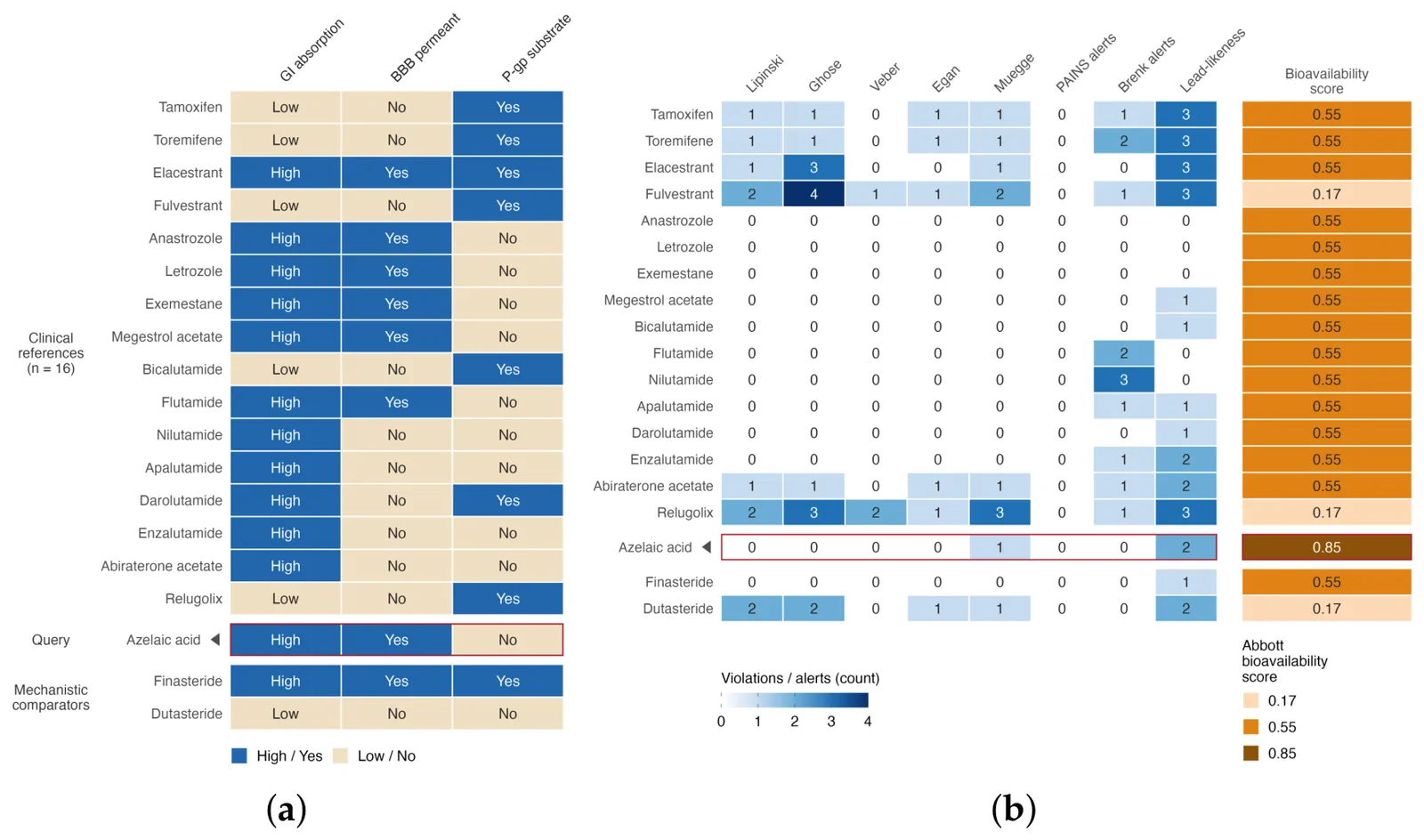
Categorical SwissADME profile of azelaic acid relative to the Clinical Reference set and Mechanistic Comparators. (**a**) Predicted absorption and disposition characteristics, including gastrointestinal absorption, blood-brain barrier permeability, and P-glycoprotein substrate status. (**b**) Drug-likeness and medicinal chemistry outputs, including Lipinski, Ghose, Veber, Egan, and Muegge rule violations, bioavailability score, pan-assay interference compound (PAINS) alerts, Brenk alerts, and lead-likeness violations. Azelaic acid is highlighted by a red outline as the Query molecule, while finasteride and dutasteride are included as projected Mechanistic Comparators. The 16 Clinical Reference compounds provide the comparative clinical context. Categorical and ordinal endpoints were analyzed separately from the continuous descriptor space and did not contribute to PCA or nearest neighbor calculations. Reported counts and percentages are descriptive frequencies within the Clinical Reference panel and do not represent inferential comparisons.

Categorical SwissADME predictions showed partial concordance between azelaic acid and the Clinical Reference set (Figure 4; Table S9). High gastrointestinal absorption was predicted for azelaic acid and for 11/16 Clinical Reference compounds (68.8%). Azelaic acid was predicted not to be a P-glycoprotein substrate, a category shared by 9 of 16 references (56.2%). By contrast, predicted blood–brain barrier permeability was positive for azelaic acid but occurred in only 6 of 16 clinical references (37.5%), with non-permeability representing the more frequent reference prediction. These values represent descriptive frequencies within the 16-compound Clinical Reference panel and were not used for inferential comparison.

Azelaic acid had no violations of the Lipinski, Ghose, Veber, or Egan rules, matching the corresponding zero-violation category in 62.5%, 62.5%, 87.5%, and 68.8% of the clinical references, respectively. It had one Muegge violation, whereas 10 of 16 references (62.5%) had none. No pan-assay interference compound (PAINS) alerts were identified for azelaic acid or for any of the 16 clinical references; this endpoint was therefore invariant and non-discriminating. Azelaic acid also had no Brenk alerts, a category observed in 7 of 16 references (43.8%) and representing the most frequent single category for this endpoint.

The SwissADME bioavailability score for azelaic acid was 0.85, while the clinical references were assigned scores of 0.55 (14/16, 87.5%) or 0.17 (2/16, 12.5%). This distinction was treated as a categorical prediction rather than as evidence of superior in vivo bioavailability. Azelaic acid had two lead-likeness violations, a category shared by 2 of 16 references (12.5%). The five cytochrome P450 inhibition endpoints were not determined for any of the 19 compounds in the SwissADME export and were therefore considered non-analyzable rather than negative predictions.

### 2.5. Extrapolative Positioning of Azelaic Acid in Standardized Physicochemical and Predicted ADME Space

In the complete eight-dimensional standardized descriptor space, the nearest Clinical Reference compound to azelaic acid was flutamide, with a Euclidean distance of 3.0063. The internal nearest neighbor distribution of the 16 clinical references yielded the 75th- and 90th-percentile thresholds of $q_{75}=1.8318$ and $q_{90}=2.6521$, respectively (Table S5).

Because the azelaic acid distance exceeded the internally defined $q_{90}$ threshold, the molecule was descriptively classified as extrapolative within this reference framework. Thus, although flutamide was the closest reference in this descriptor space, azelaic acid lay beyond the internally defined $q_{90}$ nearest-neighbor threshold of the Clinical Reference panel.

### 2.6. Robustness and Sensitivity of Reference-Space Positioning

Leave-one-reference-out analysis supported the stability of the primary classification. Azelaic acid remained extrapolative in all 16 folds. Flutamide was retained as its nearest clinical reference in 15 of 16 folds; when flutamide itself was excluded, anastrozole became the nearest reference (Table S6; Figure S4).

Bootstrap resampling provided a more graded assessment of uncertainty. Of 1000 bootstrap replicates, 992 fulfilled the prespecified validity criteria. The median azelaic acid nearest-neighbor distance across valid replicates was 3.3054 (2.5th–97.5th percentile, 2.6670–4.6744). Azelaic acid was classified as extrapolative in 73.69% of valid replicates, peripheral in 25.30%, and within the common reference neighborhood in 1.01%. Results were similar when alternative minimum numbers of distinct references were required, indicating limited sensitivity to this bootstrap specification (Table S7; Figure S5).

Additional sensitivity analyses generally supported the primary panel-relative interpretation. Replacing Consensus Log P with WLOGP retained flutamide as the nearest Clinical Reference and maintained the extrapolative classification (Table S8; Figures S6 and S7). Across the primary eight-dimensional analysis and 162 reduced-descriptor representations, the extrapolative classification remained predominant (77.30%), although nearest-reference identity was more sensitive to dimensionality. Fraction Csp3 showed the strongest influence on descriptor-level sensitivity. In PCA-reduced spaces, the classification was less stable, indicating that truncated principal-component representations did not preserve the complete local-distance geometry used in the primary analysis. Finally, disease-context sensitivity analyses retained the extrapolative classification in both the breast/endometrial and prostate subsets, although the identity of the nearest reference differed between panels.

### 2.7. Structural Positioning of Azelaic Acid Outside the Clinical Reference Neighborhood

Morgan fingerprint analysis provided an orthogonal assessment of molecular similarity. Using binary 2048-bit Morgan fingerprints with radius 2, the highest Tanimoto similarity between azelaic acid and any Clinical Reference compound was 0.08696, obtained with toremifene. This identifies toremifene only as the highest similarity reference under the specified Morgan fingerprint representation and does not indicate meaningful chemical similarity.

Importantly, structural positioning was evaluated against the nearest neighbor similarity distribution within the clinical reference set rather than against all pairwise reference similarities. Among the 16 clinical references, the minimum internal nearest neighbor Tanimoto similarity was 0.13761, while the median was 0.2600. The minimum value represents the weakest nearest-neighbor relationship observed within this specific reference panel and was used as a panel-specific comparator, not as a universal threshold for structural similarity. The maximum similarity of azelaic acid (0.08696) therefore lay below the entire internal reference nearest-neighbor distribution, corresponding to an empirical structural nearest neighbor percentile of 0%.

**Figure 5:**
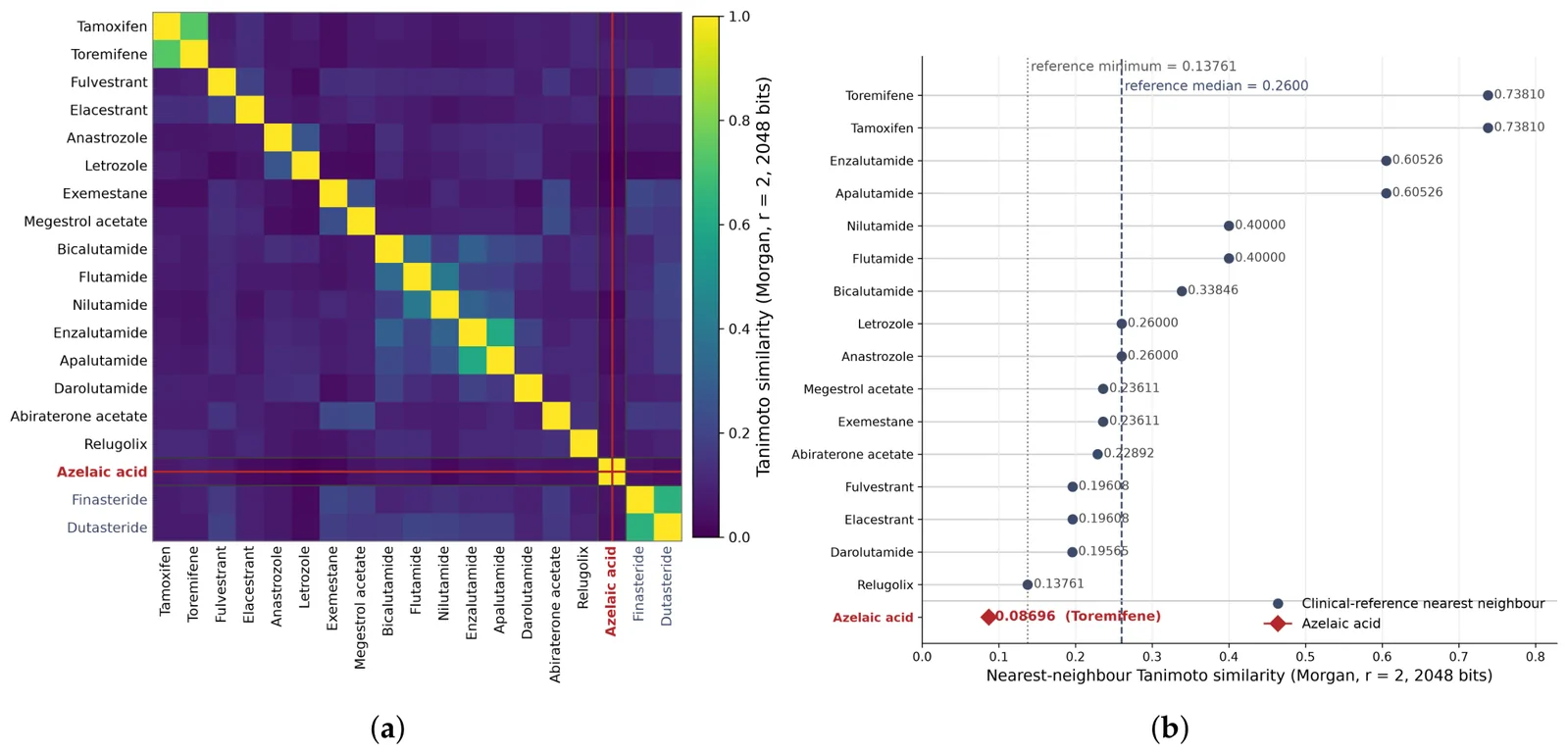
Morgan fingerprint-based structural positioning of azelaic acid relative to the Clinical Reference set. (**a**) Pairwise Tanimoto similarity matrix for the 19 study compounds using binary 2048-bit Morgan fingerprints with radius 2. Azelaic acid is highlighted as the Query molecule; finasteride and dutasteride are external Mechanistic Comparators. (**b**) Internal nearest-neighbor calibration based on the 16 Clinical Reference compounds. For each reference compound, the maximum Tanimoto similarity to any of the other 15 references is shown. The maximum similarity of azelaic acid to the reference set was 0.08696 (toremifene), below the minimum reference nearest-neighbor similarity of 0.13761 and the reference median of 0.2600.

The result was preserved when the fingerprint radius was increased from 2 to 3. Toremifene remained the highest similarity Clinical Reference, although the Tanimoto coefficient decreased to 0.06061; this value again remained below the minimum reference nearest neighbor similarity (0.11039) obtained under the radius-3 representation (Figure 5; Table S10).

Structural representation sensitivity was further assessed using MACCS keys, AtomPair fingerprints, and Topological Torsion fingerprints. Across both Morgan radii and all three additional representations, the maximum similarity of azelaic acid to the Clinical Reference panel remained below the minimum internal reference nearest-neighbor similarity of the corresponding fingerprint representation, yielding an empirical percentile of 0% in all five analyses. The Clinical Reference yielding the highest similarity varied across fingerprint families, indicating that reference identity was representation-dependent, whereas the panel-relative structural separation was preserved.

### 2.8. Integration of Physicochemical and Structural Positioning

The two similarity layers produced complementary but non-equivalent relationships. In standardized physicochemical and predicted-ADME space, flutamide was the closest clinical reference to azelaic acid, whereas toremifene yielded the highest similarity under the Morgan/Tanimoto representation. Nevertheless, azelaic acid fell outside the internally calibrated nearest neighbor range in both analyses: its physicochemical and predicted ADME distance exceeded the reference $q_{90}$ threshold, and its maximum structural similarity was lower than the nearest neighbor similarity of every clinical reference compound.

In the standardized eight-dimensional descriptor space, finasteride and dutasteride were 4.2116 and 6.0987 units from azelaic acid, respectively. Finasteride was closest to megestrol acetate within the Clinical Reference panel (d=2.2516), whereas dutasteride was closest to elacestrant (d=2.8501). Their Morgan radius-2 similarities to azelaic acid were 0.0508 and 0.0417, respectively, and their radius-3 similarities were 0.0366 and 0.0297. Their highest similarities to Clinical Reference compounds were substantially greater than their similarities to azelaic acid.

These findings indicate that the network-derived nomination of azelaic acid cannot be explained by close two-dimensional structural analogy to established endocrine anticancer agents and that the compound also occupies an extrapolative region of the reference-defined physicochemical and predicted-ADME space.

## 3. Discussion

The study compares azelaic acid with established endocrine anticancer drugs and shows that it occupies a distinct position in physicochemical and predicted ADME space and in two-dimensional structural space. In the primary standardized descriptor analysis, azelaic acid lay beyond the internally defined $q_{90}$ nearest neighbor threshold. This panel-relative classification was stable in leave-one-reference-out analysis and remained the predominant bootstrap and reduced descriptor outcome, although sensitivity analyses also showed expected dependence on descriptor representation and panel composition. Morgan fingerprint analyses independently indicated very low structural similarity to the Clinical Reference panel. These findings indicate that azelaic acid is not a close physicochemical or structural analog of the examined endocrine anticancer drugs.

The significance of this result lies not in the visually apparent structural simplicity of azelaic acid, but in converting that qualitative expectation into an internally calibrated and reproducible measure of separation. The analysis therefore quantifies how far the network-derived candidate lies from the neighborhood occupied by the prespecified clinical reference panel; its purpose is not merely to demonstrate that the molecules are chemically different.

This distinction is important for understanding the current repurposing hypothesis. Azelaic acid was prioritized in a target-based drug similarity network, where relationships between compounds relied on shared drug-target information instead of direct chemical similarities [8]. More broadly, computational drug-repositioning approaches—including network-based, structure-based, machine learning, and omics-driven strategies—are primarily intended to prioritize hypotheses for subsequent computational, experimental, or clinical validation and not to provide evidence of efficacy by themselves [4,5,6,7,8,9,10,11,37]. Related cheminformatic workflows have similarly combined drug–drug similarity analysis with computational and experimental evidence to prioritize non-obvious drug candidates [38]. The structural analysis shows that the previous network nomination cannot be explained by close two-dimensional similarity to established endocrine anticancer agents. Importantly, the absence of physicochemical or structural proximity observed here does not negate the previously reported CYP17A1 docking hypothesis; instead, it shows that this predicted target interaction is not accompanied by broad chemical or physicochemical similarity to established endocrine therapies. In this sense, azelaic acid exemplifies the non-obvious candidates generated by network-based approaches. Accordingly, the prioritization of azelaic acid does not depend on resemblance to established endocrine therapies. It is supported by the original target-based network nomination, the subsequent CYP17A1 docking hypothesis, and the broader experimental literature indicating antiproliferative and mechanistic activity. The present analysis adds a different layer of evidence by showing that this hypothesis cannot be supported through physicochemical or structural analogy to the Clinical Reference panel.

The physicochemical profile of azelaic acid helps explain this separation. Compared with the Clinical Reference panel, azelaic acid was markedly smaller, less lipophilic, more soluble according to the ESOL prediction, and less synthetically complex. At the same time, its hydrogen bonding capacity and conformational flexibility remained within the ranges occupied by established endocrine anticancer drugs. Its high Fraction Csp^3^ value further distinguished it from much of the reference panel without placing it outside the overall observed range. These characteristics should not be interpreted as intrinsically favorable or unfavorable for anticancer development, because descriptor-based ADME predictions capture broad developability trends but do not fully reproduce pharmacokinetic behavior determined by formulation, transport, metabolism, or route of administration [39]. In particular, the categorical SwissADME profile showed several properties compatible with those encountered among the clinical references, including predicted high gastrointestinal absorption, absence of PAINS alerts, and no violations of several commonly used drug-likeness rules. Conversely, the predicted bioavailability score of 0.85 should not be regarded as evidence of superior systemic exposure. Historical pharmacokinetic studies of azelaic acid demonstrated substantial dependence on formulation and route of administration, rapid decline of serum concentrations after oral administration, and extensive early urinary elimination [29,30,31,32,33,34]. These observations are especially relevant because several antiproliferative and enzyme inhibition studies used comparatively high concentrations of azelaic acid [12,17,18,19,20,21,24]. Thus, the present in silico profile identifies intrinsic molecular characteristics but does not resolve whether effective drug exposure can be achieved and maintained at tumor sites.

The endocrine context also requires cautious interpretation. Historical inhibition of 5α-reductase provides one rationale for considering azelaic acid in relation to androgen-dependent disease, and prostate and breast cancers were already proposed decades ago as possible areas for further investigation [21,24]. However, the original 5α-reductase experiments were conducted in human skin preparations and do not establish isoform-specific 5α-reductase inhibition, androgen receptor modulation, or anticancer activity in prostate tissue. Moreover, finasteride and dutasteride were deliberately included in the present analysis as external mechanistic comparators to examine whether the historical 5α-reductase association of azelaic acid is accompanied by physicochemical or structural proximity to established inhibitors of this axis. Quantitative comparison showed that neither comparator was closely positioned to azelaic acid in the standardized descriptor space or under the Morgan fingerprint representations. Their distinct positioning illustrates an important point: sharing a reported connection to the 5α-reductase axis does not imply close similarity in broader physicochemical or structural space. This does not refute the underlying biochemical observation, which requires contemporary experimental confirmation. Consequently, this analysis serves as a prioritization framework for an endocrine oncology hypothesis and not as evidence that azelaic acid shares the pharmacology of established endocrine therapies.

The structural results reinforce this interpretation from an orthogonal perspective. Fingerprint-based molecular similarity has been extensively used for compound clustering and candidate prioritization, with the Tanimoto coefficient representing a widely adopted measure for comparing molecular fingerprints [40,41,42,43,44,45]. Using this internal calibration, the maximum Tanimoto similarity of azelaic acid fell below the nearest-neighbor similarity observed for every Clinical Reference compound. The maximum reference similarity, observed with toremifene, was therefore weaker than the nearest-neighbor relationship of every reference drug within the clinical panel. The same panel-relative separation was retained across the radius-3 Morgan sensitivity analysis and the additional MACCS, AtomPair, and Topological Torsion representations. Although the identity of the highest-similarity Clinical Reference varied across fingerprint families, azelaic acid remained below the internal Clinical Reference nearest-neighbor range within every representation examined. Thus, fingerprint representation affected local neighbor identity but did not alter the panel-relative structural separation. Importantly, the nearest reference differed between the two analytical layers: flutamide in standardized physicochemical and predicted ADME space and toremifene in structural space. This divergence emphasizes that bulk molecular properties and local structural features capture different dimensions of similarity and supports their separate treatment instead of combining them into a single score. This complementary treatment of distinct analytical layers is consistent with previous work showing that methodologically different computational approaches can provide convergent evidence while retaining different inferential roles [46].

Several limitations should be considered. First, the Clinical Reference set comprised 16 compounds selected to represent major endocrine anticancer strategies and was not intended to reproduce the complete pharmacological diversity of endocrine oncology. The reference compounds were chosen independently of their SwissADME properties or similarity to azelaic acid, meaning the empirical percentiles and thresholds depend on the panel’s composition and size. The $q_{75}$/$q_{90}$ categories are therefore panel-specific empirical descriptors and should not be interpreted as universal statistical or pharmacological thresholds. The leave-one-reference-out and bootstrap analyses quantified this dependence, but they cannot replace evaluations against larger or differently defined clinical drug sets. Bootstrap sensitivity to the minimum number of distinct compounds retained per resample was limited, with the dominant classification preserved across the tested criteria; however, these results should be interpreted as resampling sensitivity within the fixed 16-compound panel, not as population-level uncertainty for endocrine anticancer drugs. Second, SwissADME outputs are structure-based predictions, not experimental pharmacokinetic data, and do not consider dose, formulation, metabolism, tissue distribution, or tumor exposure. A single prediction platform was used to maintain consistent descriptor definitions and scales across the complete reference panel. Future studies could benefit from cross-platform comparisons with alternative prediction tools, such as pkCSM or ADMETlab, along with validation against experimental pharmacokinetic data. Third, all fingerprint analyses were derived from the same two-dimensional molecular structures and should not be regarded as independent replications. Molecular size and fingerprint sparsity may contribute to low shared-bit similarity, particularly for Topological Torsion, and none of the evaluated representations assesses three-dimensional conformational similarity or target binding. Finally, no conclusion regarding anticancer efficacy, mechanism of action, or clinical utility can be drawn from the present analysis. Accordingly, the present results should be interpreted as a computational prioritization and boundary definition framework, not as evidence of pharmacological efficacy or pharmaceutical developability. The analysis does not establish target engagement, pharmacological selectivity, or whether biologically active concentrations are achievable in vivo. These unresolved questions define the scope within which the present findings can inform subsequent pharmacological and pharmaceutical development.

From a translational perspective, the present findings define a staged sequence of proposed pharmacological and pharmaceutical actions. First, the proposed CYP17A1 interaction and historical 5α-reductase effect should be re-evaluated using contemporary target-specific assays, including isoform-specific testing where appropriate, to establish whether azelaic acid produces measurable and reproducible target-related effects. Second, these findings should be examined in relevant endocrine cancer models using concentration–response and time-course experiments, pathway-level readouts, and appropriate non-malignant controls to assess pharmacological activity and selectivity. Third, experimentally active concentrations should be compared with achievable exposure through pharmacokinetic studies addressing systemic concentration, persistence, tissue distribution, and tumor exposure. If exposure proves insufficient, the next pharmaceutical step would be to evaluate whether route of administration, formulation, or delivery strategies can improve exposure at the relevant tissue site. Only if target-related activity, biological effects, and exposure feasibility are demonstrated would more advanced computer-aided drug design studies become justified. These could include structure-based refinement, analog prioritization, and optimization of potency, selectivity, and physicochemical or biopharmaceutical properties. Such experimental validation may also be complemented by appropriate analytical approaches [47].

This sequence also defines the potential practical application of the present analysis. From a pharmacological perspective, it prioritizes the mechanistic and concentration-dependent effects that must be validated before candidate advancement. From a pharmaceutical perspective, it identifies exposure, route of administration, formulation, and tissue delivery as critical development constraints. Successful repurposing trajectories of structurally simple compounds provide relevant precedent for such staged evaluation. Dimethyl fumarate, a simple fumaric acid ester historically used in psoriasis, subsequently became an effective oral therapy for relapsing multiple sclerosis [48,49]; hydroxyurea was successfully repurposed from antineoplastic use to disease-modifying treatment of sickle cell disease [50,51]. These examples do not imply mechanistic analogy or predict successful development of azelaic acid. Instead, they illustrate that molecular simplicity does not preclude clinically meaningful pharmacology in a new therapeutic context. Whether azelaic acid can follow a comparable translational trajectory will depend on the sequential pharmacological and pharmaceutical validation steps outlined above.

Within these constraints, this analysis clarifies the original network-derived hypothesis regarding azelaic acid. It is not a close chemical analogue of existing endocrine anticancer drugs but represents a distinct candidate with some developability characteristics similar to clinically used agents. This combination of network-based nomination, non-canonical chemical structure, and partially overlapping developability features supports further hypothesis-driven investigation while clearly separating computational prioritization from evidence of therapeutic efficacy.

## 4. Materials and Methods

### 4.1. Study Design and Analytical Framework

We designed this study as a comparative chemoinformatic analysis in which azelaic acid was positioned relative to a clinically anchored reference space of endocrine anticancer drugs. Nineteen small molecules were included and assigned a priori to one of three analytical roles. Sixteen clinically used endocrine anticancer agents constituted the Clinical Reference set and exclusively defined all reference distributions, centering and scaling parameters, descriptor selection, principal component axes, and empirical nearest neighbor thresholds. Azelaic acid was treated as the single Query molecule and did not contribute to the definition of the reference space. Finasteride and dutasteride were included as Mechanistic Comparator molecules because of their established inhibition of 5α-reductase and were analyzed as external comparators, not as endocrine anticancer reference drugs. They did not contribute to reference-derived centering, scaling, PCA fitting, or internal nearest-neighbor distributions; their physicochemical distances and structural similarities were calculated using the same descriptor and fingerprint representations applied to the Clinical Reference compounds.

The clinical reference panel comprised eight agents used in breast and/or endometrial cancer—tamoxifen, toremifene, fulvestrant, elacestrant, anastrozole, letrozole, exemestane, and megestrol acetate—and eight agents used in prostate cancer—bicalutamide, flutamide, nilutamide, enzalutamide, apalutamide, darolutamide, abiraterone acetate, and relugolix. The panel was assembled a priori to provide broad coverage of the major pharmacological strategies used in endocrine oncology, with equal representation of breast, endometrial and prostate cancer therapies. Its mechanistic and physicochemical diversity was intentional; the panel was not intended to constitute a homogeneous drug class or an exhaustive list of all available endocrine anticancer therapies. No reference compound was selected or excluded on the basis of its SwissADME properties or its similarity to azelaic acid. Accordingly, the Clinical Reference set was intended to define a therapeutic-context benchmark, whereas finasteride and dutasteride provided a separate mechanistic-context comparison. Details of the complete compound set, including pharmacological classes, cancer contexts, analyzed molecular entities, PubChem identifiers, SMILES strings, and InChIKeys, are provided in Table S1.

### 4.2. Molecular Structure Retrieval and Standardization

Molecular structures were retrieved and cross-checked against PubChem records [42]. For each compound, the analyzed entity was defined before any descriptor calculation, and the same structure was subsequently used throughout the SwissADME and fingerprint analyses. Single-fragment, formally neutral structures were submitted; salts and counterions associated with marketed formulations were not included.

We paid particular attention to molecular entities for which the administered compound may differ from an active metabolite or for which alternative structures could otherwise be inadvertently analyzed. Accordingly, we retained abiraterone acetate instead of abiraterone, megestrol acetate instead of megestrol, and the validated structure of toremifene. Flutamide was analyzed as the administered parent compound despite its conversion to the more potent metabolite 2-hydroxyflutamide. The molecular form used for each compound and relevant entity-specific considerations are documented in Table S1. Structural identity was verified by concordance of the submitted structures with PubChem identifiers and InChIKeys before analysis.

### 4.3. SwissADME Analysis and Data Processing

All 19 compounds were submitted in a single batch to SwissADME [35], and the resulting CSV export was retained as the primary raw dataset. SwissADME was the sole source of the physicochemical, predicted pharmacokinetic, drug-likeness, and medicinal chemistry variables used in the reference space analyses.

Ten continuous descriptors were prespecified as candidates for quantitative analysis: molecular weight (MW), molar refractivity (MR), topological polar surface area (TPSA), number of hydrogen bond acceptors (HBA), number of hydrogen bond donors (HBD), number of rotatable bonds, fraction of sp^3^ carbons (Fraction Csp^3^), Consensus Log P, ESOL Log S, and synthetic accessibility score. Consensus Log P was selected as the primary lipophilicity descriptor, while WLOGP was reserved for a prespecified sensitivity analysis. In the present SwissADME export, iLOGP was not determined for any of the 19 molecules; Consensus Log P was therefore used exactly as returned by SwissADME and corresponded numerically to the consensus of the four available component estimates (XLOGP3, WLOGP, MLOGP, and Silicos-IT Log P).

Categorical or ordinal SwissADME outputs considered separately from the continuous descriptor analyses included predicted gastrointestinal absorption, blood–brain barrier permeability, P-glycoprotein substrate status, Lipinski, Ghose, Veber, Egan, and Muegge rule violations, bioavailability score, PAINS alerts, Brenk alerts, and lead-likeness violations. The five CYP inhibition fields (CYP1A2, CYP2C19, CYP2C9, CYP2D6, and CYP3A4) were returned as not determined for all 19 compounds and were therefore not used for comparative inference.

The raw CSV file was imported directly into R. Variables expected to be numeric were type-checked before numeric conversion; molecule identifiers were matched against the prespecified metadata table; and uniqueness, completeness, analytical role assignments, and the expected panel size of 19 molecules were verified before any statistical analysis.

### 4.4. Univariate Reference Space Positioning

Reference distributions were calculated exclusively from the 16 Clinical Reference compounds. For each continuous descriptor, the mean, standard deviation, median, interquartile range, minimum, and maximum were calculated. Azelaic acid, finasteride, and dutasteride were subsequently positioned against these fixed reference distributions. For a projected molecule with descriptor value *x*, the empirical percentile was calculated according to Equation (1):

(1) $$P=100\times \frac{N\left(x_{i}<x\right)+0.5N\left(x_{i}=x\right)}{16},$$

where $x_{i}$ denotes the corresponding descriptor values for the 16 clinical reference compounds, and *N* denotes the number of reference compounds satisfying the specified condition.

Reference standardized scores were calculated with Equation (2)

(2) $$z=\frac{x-{\bar{x}}_{ref}}{s_{ref}},$$

where ${\bar{x}}_{ref}$ and $s_{ref}$ are the mean and sample standard deviation of the clinical reference set.

Each projected value was additionally classified as lying within the reference interquartile range, between the interquartile range and the observed reference limits, or outside the reference range. No inferential test was applied to compare a single query molecule with the clinical reference group.

### 4.5. Descriptor Screening and Redundancy Reduction

Descriptor selection was performed exclusively on the 16 Clinical Reference compounds; azelaic acid, finasteride, and dutasteride were excluded from all screening and selection steps. The ten candidate descriptors were first screened for insufficient variation. A descriptor was prespecified for removal if it contained fewer than three distinct values among the 16 references or if a single modal value represented at least 90% of the reference observations. None of the ten candidates met these exclusion criteria.

Pairwise correlation screening was used to reduce redundant molecular descriptors before multivariate analysis [52]. Hence, pairwise Spearman rank correlations were then calculated across the reference compounds. Descriptor pairs with an absolute correlation coefficient |ρ|>0.85 were considered redundant. Redundant pairs were processed in descending order of |ρ|; within each pair, the descriptor with the larger mean absolute correlation with the remaining descriptors was removed. A prespecified descriptor order was used only to resolve exact ties.

Application of this procedure removed molar refractivity and TPSA and retained eight descriptors for the primary multivariate analysis: MW, HBA, HBD, rotatable bonds, Fraction Csp^3^, Consensus Log P, ESOL Log S, and synthetic accessibility score [52]. The complete Spearman correlation matrix and descriptor elimination log are provided in Tables S2 and S3, and Figure S1.

### 4.6. Reference-Only Principal Component Analysis

Principal component analysis (PCA) was performed on the eight retained continuous descriptors using only the 16 Clinical Reference compounds. Each descriptor was centered and scaled using the reference set mean and sample standard deviation before decomposition. Azelaic acid, finasteride, and dutasteride were then projected into the resulting PCA model as supplementary observations using the same reference-derived centering and scaling parameters. Consequently, none of the three projected molecules influenced the component axes, loadings, or explained variance.

PCA was used as a descriptive ordination of the reference-defined multidimensional space [53]. The first two principal components were used for visualization, while all component loadings and explained variance values were retained for supplementary reporting. PCA coordinates were not used to calculate molecular proximity or nearest neighbor classifications.

### 4.7. Nearest Neighbor Analysis in Standardized Descriptor Space

Quantitative proximity to the clinical reference set was assessed in the complete eight-dimensional standardized descriptor space, rather than in the two-dimensional PCA projection. Euclidean distances were calculated after applying the reference-derived centering and scaling parameters to all molecules.

To establish an empirical internal reference distribution, each of the 16 Clinical Reference compounds was compared with the remaining 15 reference compounds, and its smallest Euclidean distance was retained as its internal nearest neighbor distance. The resulting 16 distances were used to calculate the median and the 75th and 90th percentiles ($q_{75}$ and $q_{90}$), using the type 7 sample quantile estimator [54]. The $q_{75}$ and $q_{90}$ thresholds were prespecified as descriptive empirical cutoffs, not as inferential or pharmacological decision limits. The $q_{75}$ threshold identifies the upper quartile of internal nearest-neighbor distances, whereas $q_{90}$ denotes the upper tail of the observed reference distribution without imposing a parametric distributional model. These thresholds are specific to the present reference panel and require recalibration if the panel composition changes.

For each projected molecule, the nearest clinical reference and corresponding Euclidean distance *d* were identified. Relative position was classified using prespecified empirical thresholds:

- $d\leq q_{75}$: common reference neighborhood;
- $q_{75}<d\leq q_{90}$: peripheral;
- $d>q_{90}$: extrapolative.

These categories are descriptive, panel-specific labels that characterize position relative to the observed physicochemical or predicted ADME reference space and they do not represent formal statistical classes, pharmacological categories, or efficacy classifications. Distances to all 16 reference compounds and the margin relative to the governing empirical threshold were retained for reporting.

### 4.8. Robustness and Sensitivity Analyses

Two resampling analyses evaluated whether the positioning of azelaic acid depended strongly on the composition of the relatively small clinical reference panel. The eight-descriptor set was held fixed so that changes in positioning reflected changes in the reference compounds and not repeated feature selection.

As a post hoc disease-context sensitivity analysis, the nearest neighbor analysis was repeated separately within the eight breast/endometrial agents and the eight prostate cancer agents. The eight retained descriptors were held fixed, while centering, scaling, internal nearest neighbor distances, and $q_{75}/q_{90}$ thresholds were recalculated independently within each subset using the same procedures as in the primary analysis.

Dimensionality sensitivity was evaluated without repeating descriptor selection. Starting from the fixed primary set of eight retained descriptors, we performed eight leave-one-descriptor-out analyses and exhaustively evaluated all 28 six-descriptor, 56 five-descriptor, and 70 four-descriptor combinations. For each representation, reference-derived centering and scaling, internal nearest-neighbor distances, $q_{75}$ and $q_{90}$ thresholds, nearest-reference identity, and empirical classification were recalculated. Separately, PCA was fitted only to the 16 standardized Clinical Reference compounds, azelaic acid was projected as a supplementary observation, and distances and thresholds were recalculated independently in PC1–PC2, PC1–PC3, and PC1–PC4 spaces.

#### 4.8.1. Leave-One-Reference-Out Analysis

A leave-one-reference-out (LORO) analysis was performed in 16 folds. In each fold, one clinical reference compound was removed, and all downstream quantities were recalculated using the remaining 15 references, including centering and scaling parameters, the PCA model, internal nearest neighbor distances, $q_{75}$ and $q_{90}$ thresholds, and the nearest neighbor distance and classification of azelaic acid. The identity of the nearest reference and the resulting empirical classification were recorded for each fold.

#### 4.8.2. Bootstrap Analysis

A nonparametric bootstrap with 1000 replicates was performed by sampling 16 clinical reference compounds with replacement. Within each replicate, centering and scaling parameters were recalculated from the sampled reference observations, with their bootstrap multiplicities retained. A replicate was considered valid if at least eight distinct clinical references were represented and all eight retained descriptors had a non-zero standard deviation. This eight-reference requirement was used as a pragmatic safeguard against highly degenerate resamples and was not treated as a formal statistical threshold. As a sensitivity analysis, the same 1000 bootstrap draws were re-evaluated using minimum requirements of 6, 8, and 10 distinct Clinical Reference compounds. All other analytical steps were unchanged. Bootstrap multiplicities were retained for replicate-specific centering, scaling, and standardization, whereas duplicate copies of the same molecule were collapsed only when constructing the internal nearest-neighbor distribution to avoid artificial zero distances.

Because resampling with replacement creates duplicate copies of the same molecule and therefore artificial zero nearest neighbor distances, the primary bootstrap $q_{75}$ and $q_{90}$ thresholds were calculated from the distinct molecular entities represented in each valid replicate. The query molecule was standardized using the replicate-specific reference parameters, and its nearest clinical reference, Euclidean distance, $q_{75}$, $q_{90}$, and empirical classification were recorded. Invalid replicates were retained in the raw bootstrap file but excluded from summary proportions. The median bootstrap distance, 2.5th–97.5th percentile interval, nearest neighbor frequencies, and proportions assigned to each empirical neighborhood category were calculated across valid replicates. A fixed random seed (20260729) was used for bootstrap resampling to ensure computational reproducibility.

#### 4.8.3. Lipophilicity Sensitivity Analysis

To assess dependence on the lipophilicity estimator, the primary multivariate workflow was repeated after replacing Consensus Log P with WLOGP. Descriptor redundancy screening, reference-only PCA, reference standardization, nearest neighbor distances, and empirical $q_{75}$/$q_{90}$ classification were recalculated under this alternative parameterization. The sensitivity analysis was interpreted based on the stability of the nearest neighbor identity and empirical classification instead of according to small numerical differences between the two models.

### 4.9. Categorical ADME, Drug-Likeness, and Medicinal Chemistry Profiles

Categorical and ordinal SwissADME outputs were analyzed separately from the continuous descriptor space and did not enter the PCA or Euclidean distance calculations. For each analyzable endpoint, the number and percentage of the 16 Clinical Reference compounds belonging to each category were calculated, and the corresponding prediction for azelaic acid was reported relative to this reference frequency. Finasteride and dutasteride were treated analogously as externally projected comparators. These frequencies were reported descriptively; no inferential statistical comparison was performed between the single Query molecule and the Clinical Reference panel.

Endpoints invariant across all 19 molecules were retained descriptively but were not interpreted as discriminating features. In particular, pan-assay interference compound (PAINS) alerts were absent for all compounds in the panel. Endpoints deemed non-analyzable for all molecules, including the five CYP inhibition fields, were not coded as negative predictions.

### 4.10. Morgan Fingerprints and Tanimoto Structural Similarity

Two-dimensional structural similarity was evaluated independently of the SwissADME descriptor space using RDKit. Morgan fingerprints were generated using the RDKit rdFingerprintGenerator.GetMorganGenerator interface (RDKit 2026.03.4), with radius=2 and fpSize=2048, corresponding to an ECFP4-like representation [40,41]. Binary bit vectors were obtained using GetFingerprint(). Standard Morgan connectivity invariants and default bond invariants were used, with bond-type information included and chirality not encoded; no custom atom or bond invariants were supplied. Molecules were generated from SMILES using standard RDKit sanitization and aromaticity perception. Tanimoto similarities were calculated using DataStructs.BulkTanimotoSimilarity.

Pairwise structural similarity was quantified using the Tanimoto coefficient [43], generating a symmetric 19×19 similarity matrix. For azelaic acid, similarity to each of the 16 clinical references was ranked, and the maximum reference similarity was identified. Finasteride and dutasteride were analyzed in the same manner.

Structural positioning was calibrated against the internal similarity structure of the clinical panel. For each of the 16 Clinical Reference compounds, the maximum Tanimoto similarity to any of the other 15 clinical references was calculated, producing a 16-value reference nearest neighbor similarity distribution. The maximum similarity of azelaic acid to the clinical reference set was then located relative to this distribution using the same empirical percentile convention as in the univariate analysis. This approach compares like with like—a query-to-nearest reference similarity with the nearest neighbor similarity ordinarily observed within the clinical reference set—rather than comparing a maximum query similarity with the distribution of all possible reference–reference pairs.

No universal Tanimoto threshold for structural similarity was imposed, and structural similarity was not combined with SwissADME-based distance into a composite score [43,45]. The two measures were interpreted as complementary: Morgan and Tanimoto quantify shared local two-dimensional atomic environments, whereas the standardized SwissADME distance quantifies similarity in bulk physicochemical and predicted ADME properties.

As a prespecified sensitivity analysis, the structural similarity calculation was repeated using Morgan fingerprints with a radius of 3 (ECFP6-like representation) and the same 2048-bit length. Stability was evaluated from the identity of the highest similarity Clinical Reference and the position of azelaic acid relative to the reference nearest neighbor similarity distribution.

As an additional sensitivity analysis of molecular representation, the 19 validated structures were encoded using MACCS keys, 2048-bit hashed binary AtomPair fingerprints, and 2048-bit hashed binary Topological Torsion fingerprints in RDKit 2026.03.4. AtomPair and Topological Torsion fingerprints were generated as bit vectors with count simulation disabled. For each representation, the maximum Tanimoto similarity of azelaic acid to the 16 Clinical Reference compounds was compared with the 16 internal reference nearest-neighbor similarities defined within the same fingerprint representation. Tanimoto values from different fingerprint families were not compared numerically.

### 4.11. Software, Reproducibility, and Data Availability

Data processing, descriptive statistics, descriptor screening, PCA, nearest neighbor analysis, leave-one-reference-out analysis, bootstrap resampling, and graphical outputs were implemented in R version 4.5.3. The analysis pipeline was organized as sequential scripts executed from a single entry point, with analytical roles, descriptor definitions, thresholds, quantile method, and random seed declared centrally before execution. The main data-handling and visualization workflow used the tidyverse ecosystem and ggplot2.

Morgan fingerprints and Tanimoto similarities were calculated in Python 3.13.5 using RDKit version 2026.03.4. Fingerprint generation and pairwise Tanimoto calculations were deterministic and independent of random-number generation. The fixed random seed specified above was used for bootstrap resampling; stochastic graphical jitter, where applied, affected visualization only and not numerical results.

The complete molecular structure table, original SwissADME CSV export, processed descriptor matrices, correlation and descriptor-selection outputs, PCA scores and loadings, distance matrices, LORO and bootstrap results, structural similarity matrices, analysis scripts, software versions, and random number provenance were archived with the study to permit full computational reproduction.

## 5. Conclusions

Azelaic acid occupies a distinct position relative to established endocrine anticancer drugs represented by the prespecified 16-drug Clinical Reference panel in physicochemical and predicted ADME and in two-dimensional structural space. Although flutamide was its nearest Clinical Reference in the standardized descriptor space, azelaic acid lay beyond the internally defined $q_{90}$ nearest-neighbor threshold in the primary analysis, with this classification remaining dominant, but not invariant, under bootstrap resampling. This panel-relative separation was also retained in both disease-context subsets and remained the predominant outcome across reduced-descriptor sensitivity analyses, although it was not invariant to dimensionality or representation. Across the evaluated two-dimensional fingerprint representations, structural similarity remained below the internal Clinical Reference nearest-neighbor range. At the same time, several predicted ADME and drug-likeness characteristics overlapped with those of clinically used agents. These findings refine the original network-derived repurposing hypothesis by identifying azelaic acid as a non-canonical candidate, not a close analog of existing endocrine therapies. Further prioritization should therefore focus on experimental validation of previously proposed mechanistic hypotheses and on exposure feasibility, not on chemical extrapolation from established endocrine therapies. The results support further hypothesis-driven investigation while underscoring that the present findings are specific to the reference panel and analytical representations examined and that computational positioning alone cannot establish anticancer efficacy, mechanism of action, or clinical utility. The practical value of this analysis lies in guiding pharmacological and pharmaceutical development. It identifies which mechanistic hypotheses require validation, which concentration and exposure constraints must be resolved, and when formulation, delivery, or structure-optimization strategies become scientifically justified. In this framework, progression from computational prioritization to candidate development requires sequential evidence of target-related activity, relevant cellular effects, pharmacological selectivity, and achievable exposure before pursuing advanced formulation or analog-development strategies.

## Figures and Tables

**Table 1:** Univariate physicochemical and predicted ADME positioning of azelaic acid across the eight retained descriptors relative to the Clinical Reference set.

Descriptor	Azelaic Acid	Reference Min–Max	Reference IQR	Empirical Percentile	*z*-Score	Position
MW (g/mol)	188.22	276.21–623.63	312.02–460.08	0.0%	−2.07	Below range
HBA	4	2–10	3.00–7.25	40.6%	−0.47	Within IQR
HBD	2	0–3	0.00–2.00	81.3%	1.07	Within IQR
Rotatable bonds	8	0–15	3.00–8.25	71.9%	0.49	Within IQR
Fraction Csp^3^	0.78	0.06–0.81	0.23–0.46	93.8%	1.96	Above IQR, within range
Consensus Log P	1.50	1.61–8.75	2.49–5.33	0.0%	−1.25	Below range
ESOL Log S	−1.47	−8.53 to −3.04	−6.43 to −3.60	100%	2.05	Above range
Synthetic accessibility	1.57	2.05–5.98	2.84–4.72	0.0%	−1.74	Below range

Abbreviations: IQR, interquartile range; MW, molecular weight; HBA, hydrogen bond acceptor; HBD, hydrogen bond donor; ESOL Log S, estimated solubility.

## Data Availability

The original contributions presented in this study are included in the article and Supplementary Materials. Further inquiries can be directed to the corresponding authors.

## Supplementary Materials

The following supporting information can be downloaded at: https://www.mdpi.com/article/10.3390/ph19101634/s1, Figure S1: Spearman correlation matrix of the ten candidate continuous descriptors across the 16 Clinical Reference compounds; Figure S2: Explained variance of the reference-only principal component analysis; Figure S3: Descriptor loadings on the first two components of the reference-only principal component analysis; Figure S4: The extrapolative classification of azelaic acid survives every leave-one-reference-out fold; Figure S5: Resampling the reference set leaves azelaic acid outside the reference neighborhood in the large majority of replicates; Figure S6: Sensitivity of azelaic acid positioning to the choice of lipophilicity descriptor; Figure S7: Lipophilicity-solubility positioning of azelaic acid using WLOGP in the sensitivity analysis; Table S1: Compound set, analytical roles, and molecular identities used in the study; Table S2: Spearman rank-correlation matrix of the ten candidate continuous descriptors across the 16 Clinical Reference compounds; Table S3: Descriptor elimination log following correlation-based redundancy screening in the Clinical Reference set; Table S4: Explained variance and descriptor loadings for the reference-only principal component analysis; Table S5: Nearest-neighbour positioning of azelaic acid relative to the Clinical Reference set in the eight-dimensional standardized physicochemical and predicted ADME descriptor space; Table S6: Leave-one-reference-out robustness analysis of azelaic acid positioning in the standardized physicochemical and predicted ADME descriptor space; Table S7: Bootstrap robustness analysis of azelaic acid positioning in the standardized physicochemical and predicted ADME descriptor space; Table S8: Sensitivity analysis of azelaic acid positioning after replacement of Consensus Log P with WLOGP; Table S9: Complete categorical and ordinal SwissADME profile of the 19 compounds included in the analysis; Table S10: Morgan fingerprint–based structural positioning of azelaic acid relative to the Clinical Reference set.

## Abbreviations

The following abbreviations are used in this manuscript:

ADME	Absorption, distribution, metabolism, and excretion
ECFP	Extended-connectivity fingerprint
ESOL	Estimated solubility
HBA	Hydrogen bond acceptor
HBD	Hydrogen bond donor
LORO	Leave-one-reference-out
MR	Molar refractivity
MW	Molecular weight
PAINS	Pan-assay interference compounds
PCA	Principal component analysis
P-gp	P-glycoprotein
TPSA	Topological polar surface area
WLOGP	Wildman–Crippen log P
