# Supplementary material for: Physicochemical, Predicted ADME, and Structural Positioning of Azelaic Acid Within an Endocrine Oncology Reference Framework
Source: Pharmaceuticals (Basel). 2026 Oct 15;19(10):1634. doi: 10.3390/ph19101634 (PMC13708322; doi:10.3390/ph19101634)
Supplement: Supplementary file 1 [file pharmaceuticals-19-01634-s001.zip › pharmaceuticals-4569408-supplementary.pdf]

Physicochemical, Predicted ADME, and Structural Positioning of Azelaic Acid Within an Endocrine Oncology Reference Framework

Elena-Daniela Jurj, Adrian Voicu, Daiana Colibășanu, Mirela Voicu, Mirela Nicolov, and Lucreția Udrescu

Supplementary Figures

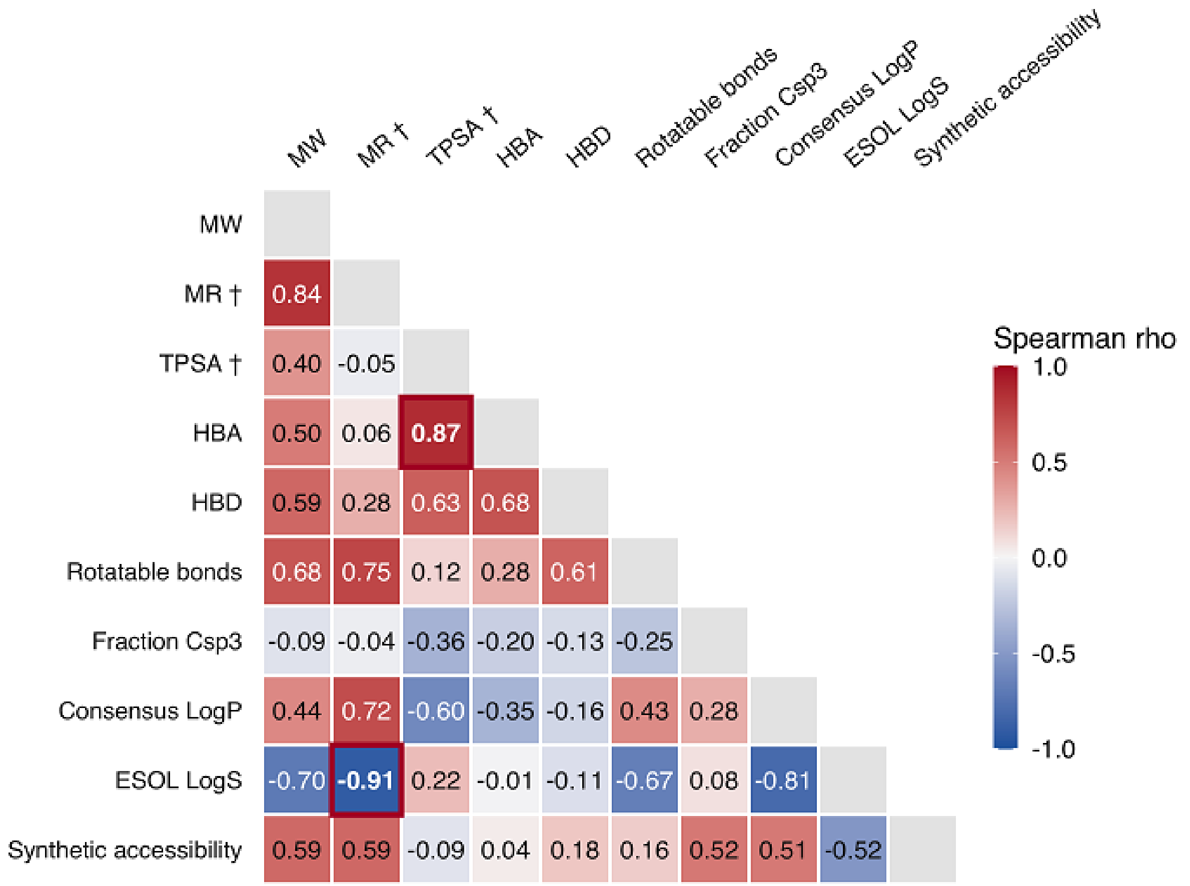

Figure S1. Spearman correlation matrix of the ten candidate continuous descriptors across the 16 Clinical Reference compounds. Pairwise correlations were calculated within the Clinical Reference set. Red outlines indicate descriptor pairs exceeding the prespecified redundancy threshold of |ρ|>0.85: MR-ESOL Log S (ρ=−0.9118) and TPSA-HBA (ρ=0.8716). MR and TPSA, indicated by †, were removed according to the prespecified redundancy-elimination procedure. The largest remaining absolute pairwise Spearman correlation among the eight retained descriptors was |ρ|=0.8118.

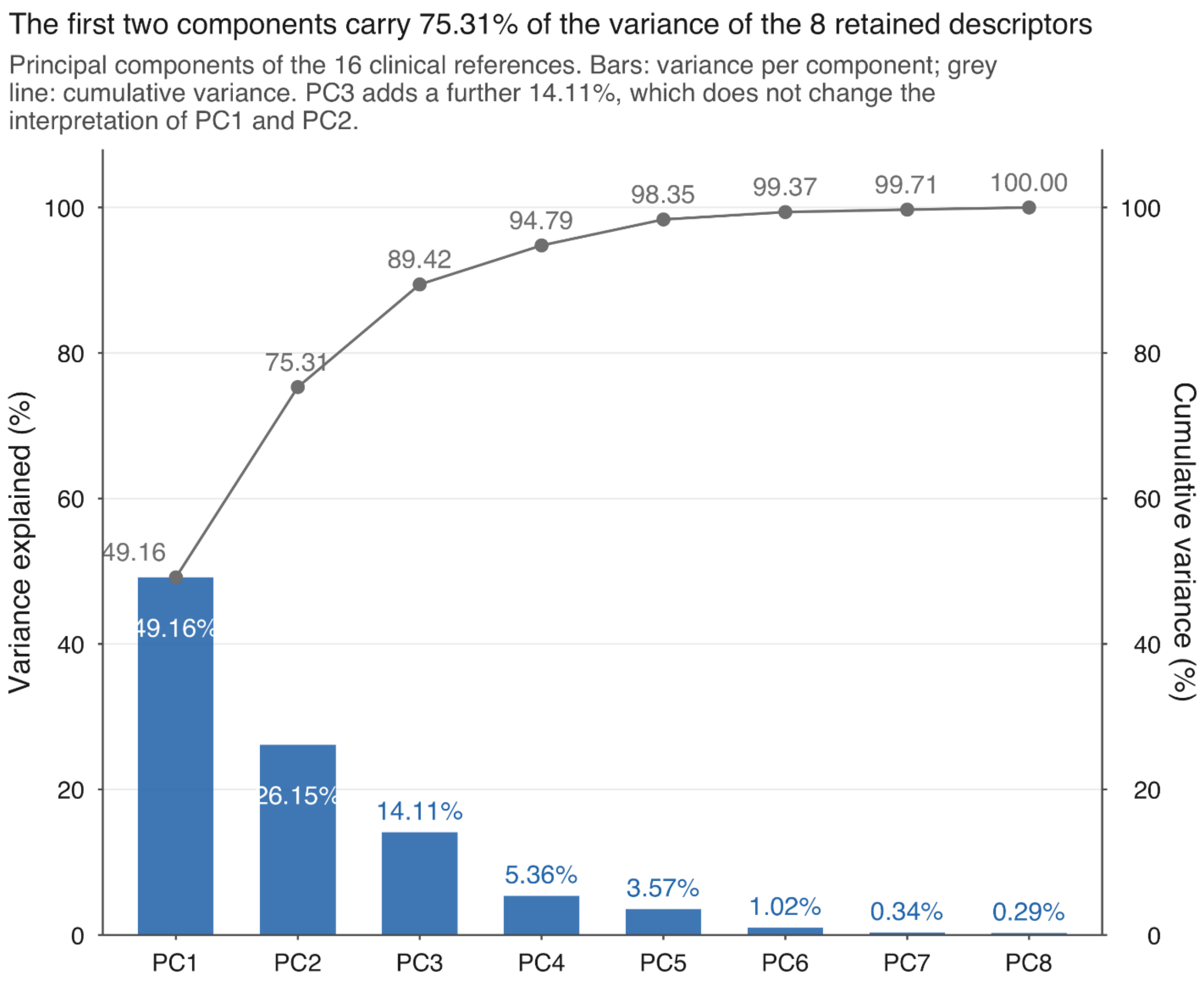

Figure S2. Explained variance of the reference-only principal component analysis. PCA was fitted exclusively to the 16 Clinical Reference compounds using the eight retained standardized descriptors. PC1 and PC2 explained 49.16% and 26.15% of the reference variance, respectively (75.31% cumulative), while PC3 accounted for an additional 14.11%. PCA was used for descriptive visualization, whereas nearest-neighbor analyses were performed in the complete eight-dimensional standardized descriptor space.

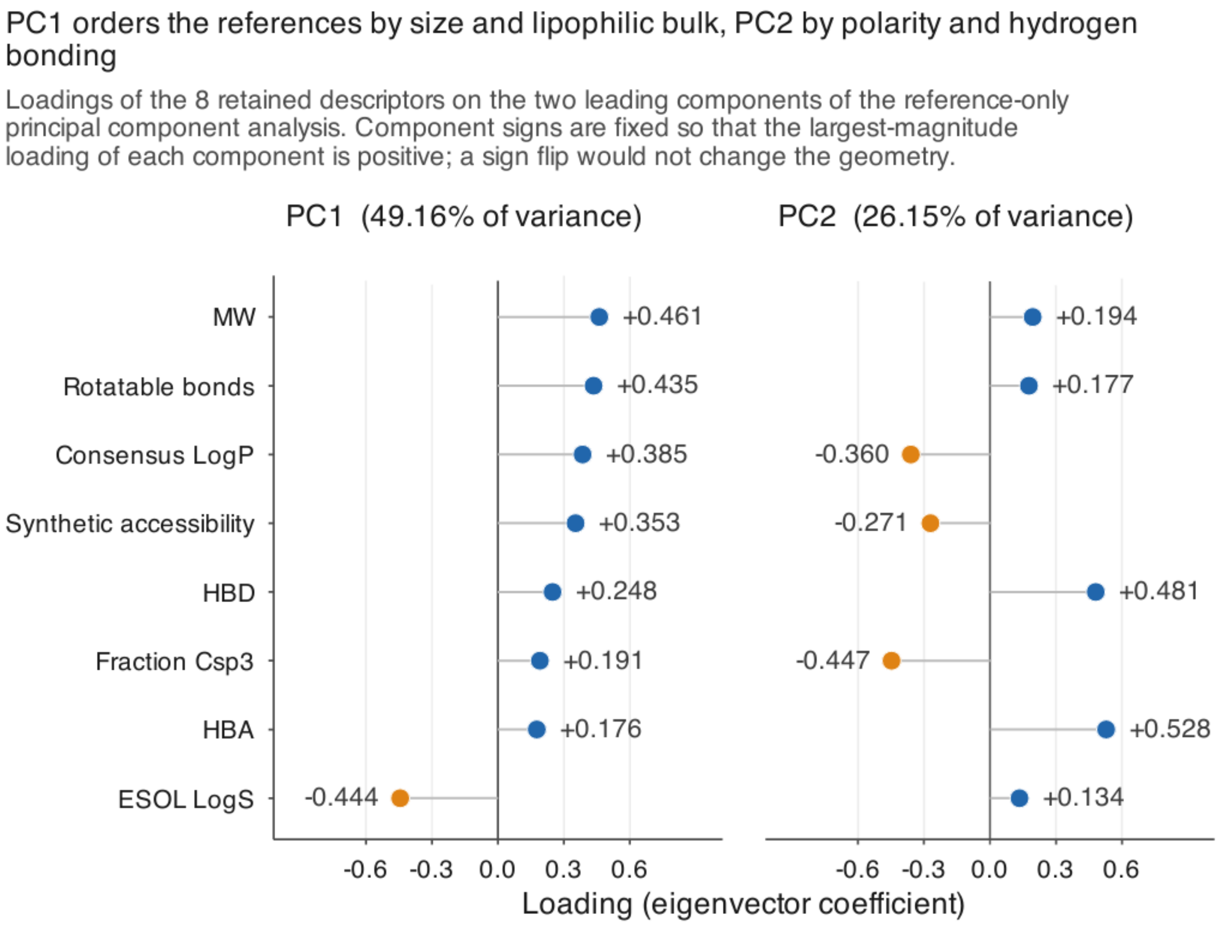

Figure S3. Descriptor loadings on the first two components of the reference-only principal component analysis. The loading pattern of PC1 primarily reflects molecular size and the lipophilicity–solubility dimension, whereas PC2 is more strongly associated with hydrogen bonding capacity, carbon saturation, and lipophilicity. Component signs correspond to the orientation of the reported PCA solution; simultaneous sign reversal of a component and its scores would not alter the underlying geometry.

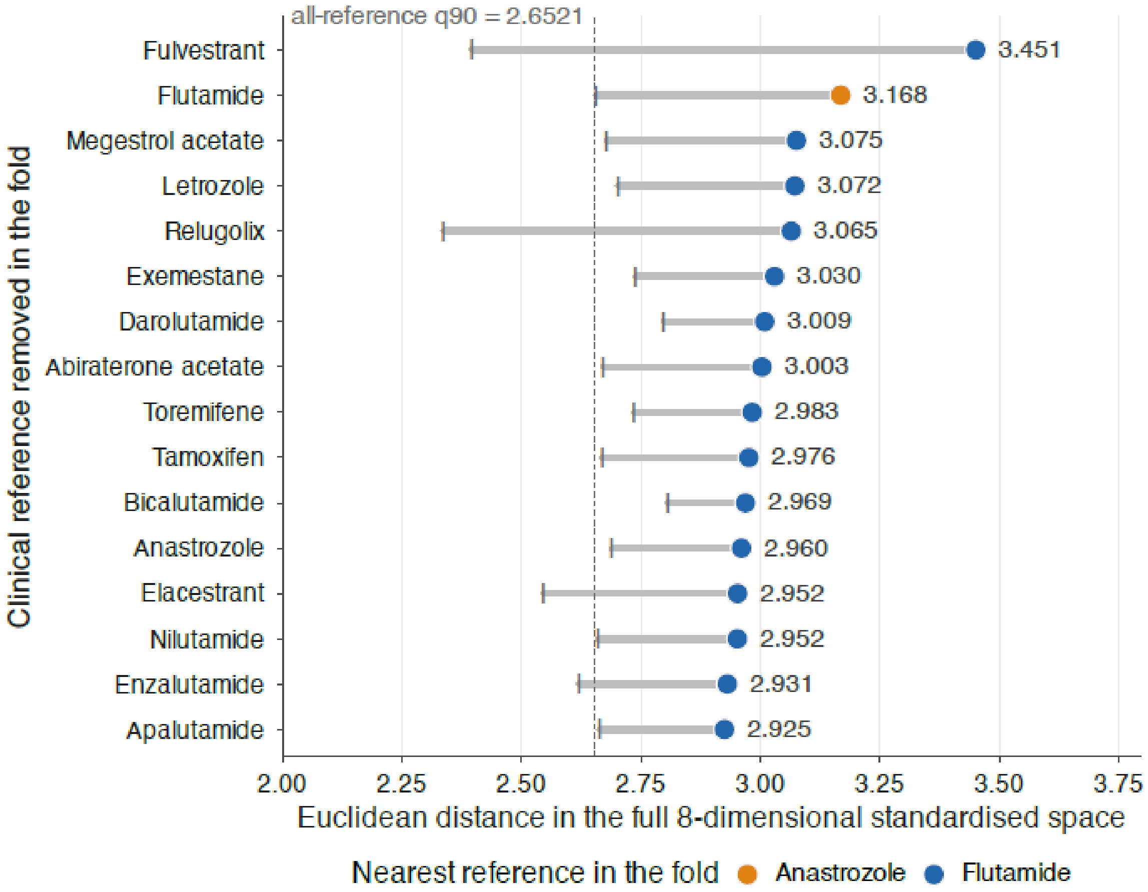

Figure S4. The extrapolative classification of azelaic acid survives every leave-one-reference-out fold. Bar: from the fold's own 90th-percentile threshold (tick) to the azelaic acid nearest neighbor distance (dot), for each of the 16 folds. The distance exceeds the threshold in 16 of 16 folds, so the classification is extrapolative throughout. The nearest Clinical Reference was flutamide in 15 of 16 folds; when flutamide itself was removed, anastrozole became the nearest reference.

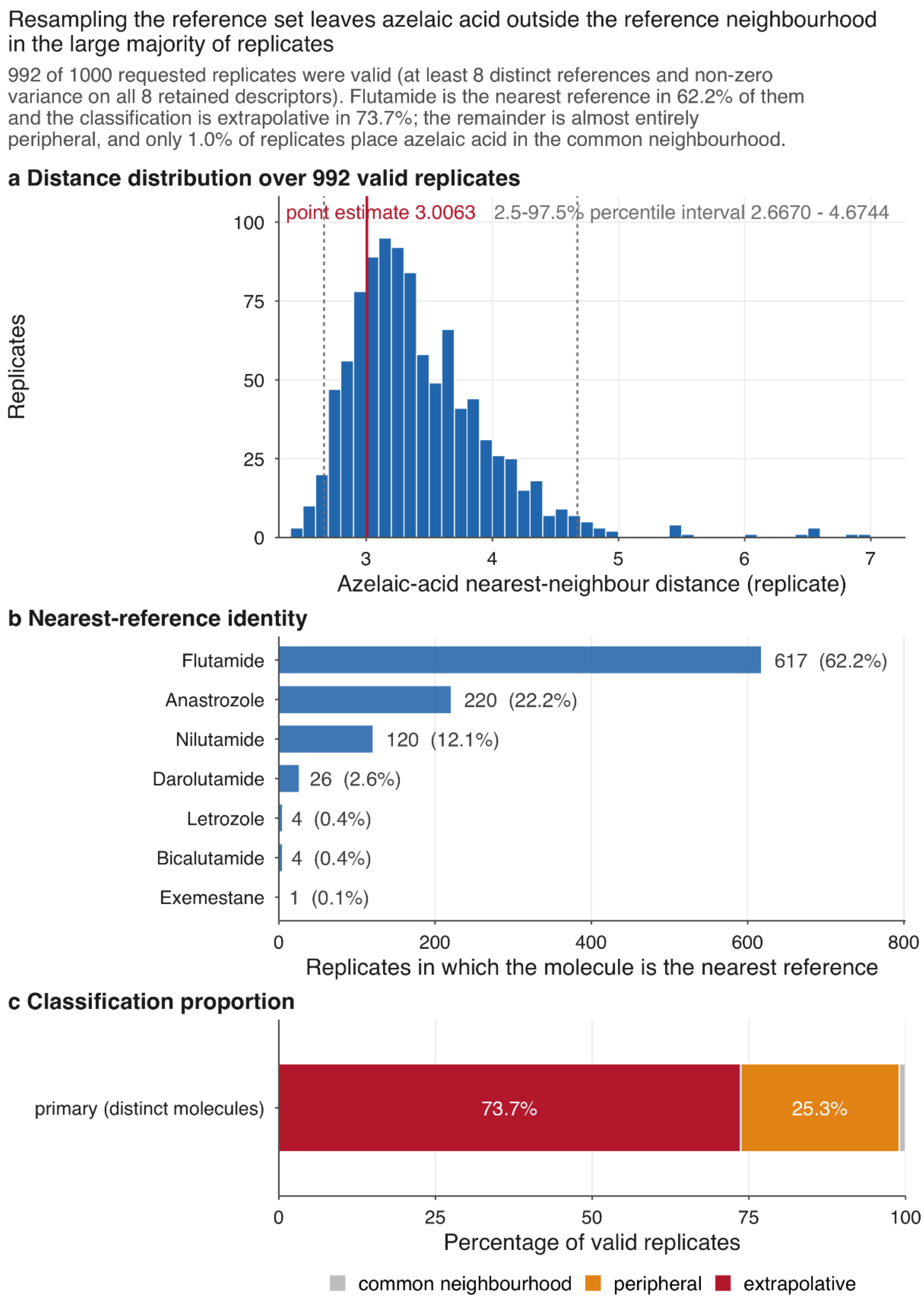

Figure S5. Resampling the reference set leaves azelaic acid outside the reference neighborhood in the large majority of replicates. 992 of 1000 requested replicates were valid (at least 8 distinct references and non-zero variance across the fixed eight-descriptor set). Flutamide is the nearest reference in 62.2% of them and the classification is extrapolative in 73.7%; the remainder is almost entirely peripheral, and only 1.0% of replicates place azelaic acid in the common neighborhood. Reference nearest neighbor thresholds were calculated from the distinct molecular entities represented in each valid replicate to prevent duplicate copies of the same molecule from introducing artificial zero nearest neighbor distances.

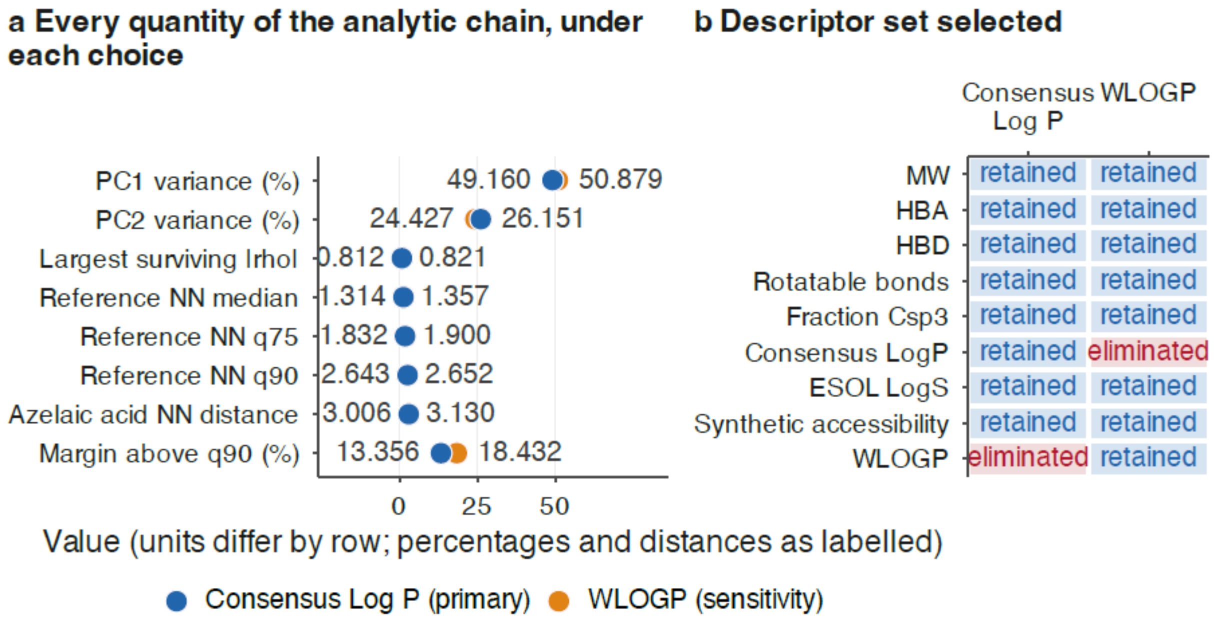

Figure S6. Sensitivity of azelaic acid positioning to the choice of lipophilicity descriptor. The analytical workflow was rerun after replacing Consensus Log P with WLOGP, while all other analytical specifications were retained. Panel (a) compares outputs from the primary and sensitivity analyses, including explained variance, the largest remaining absolute Spearman correlation after redundancy screening, internal nearest neighbor reference statistics, the azelaic acid nearest neighbor distance, and the margin above q_{90}. Panel (b) shows the descriptor sets retained after redundancy screening. In both analyses, flutamide remained the nearest Clinical Reference to azelaic acid, which continued to be classified as extrapolative relative to the Clinical Reference space.

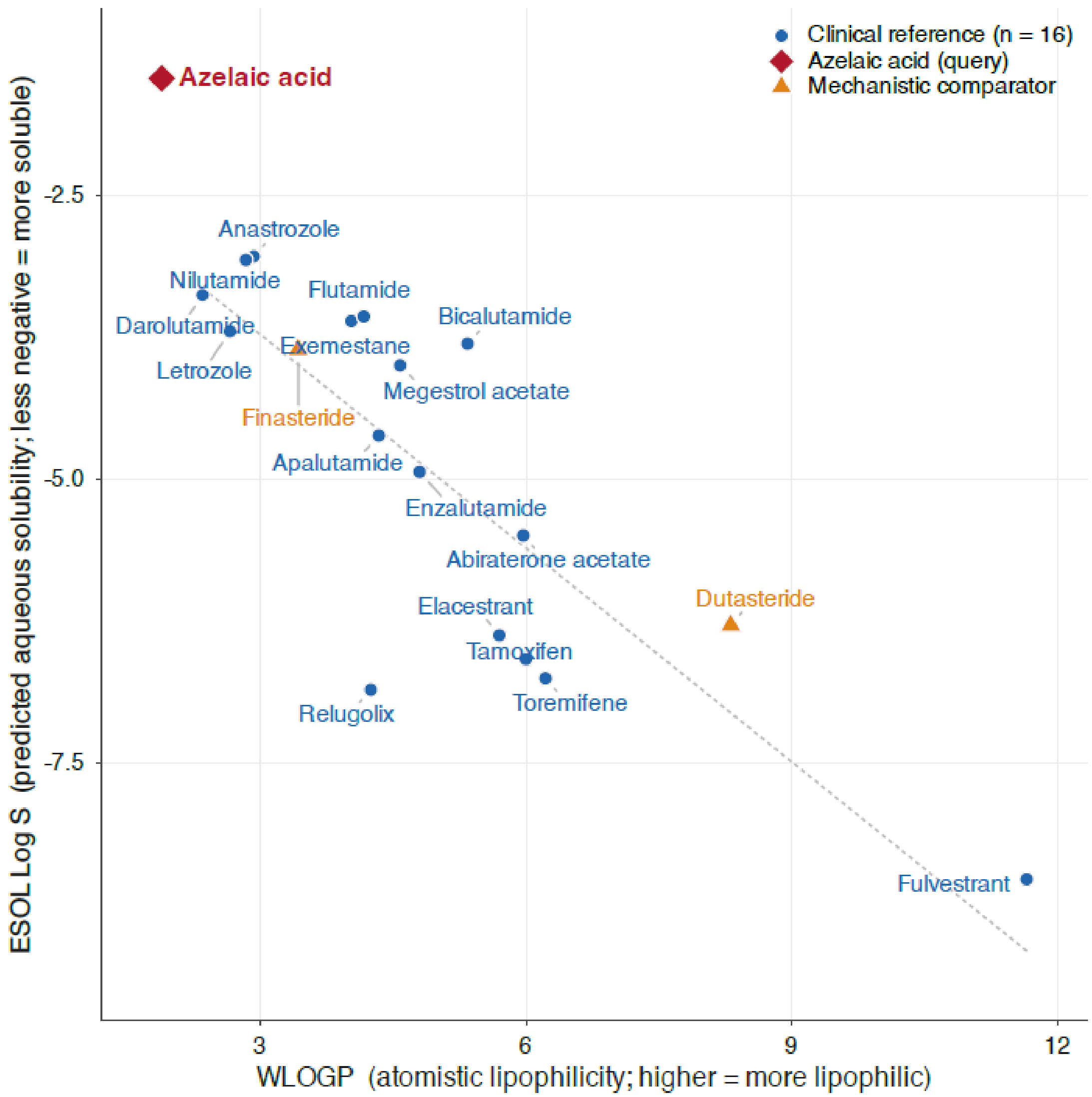

Figure S7. Lipophilicity-solubility positioning of azelaic acid using WLOGP in the sensitivity analysis. Across the 16 Clinical Reference compounds, WLOGP and ESOL Log S were strongly inversely related (Spearman $\rho = -0.821$). Azelaic acid occupied the low WLOGP, high-predicted solubility region beyond the corresponding values of all Clinical Reference compounds. The dashed line represents the ordinary least-squares fit to the 16 Clinical Reference compounds only. Finasteride and dutasteride are shown as external Mechanistic Comparators.
